# 1,5-Bis(penta­fluoro­phen­yl)-3-phenyl­pent-2-ene-1,5-dione

**DOI:** 10.1107/S1600536809041701

**Published:** 2009-10-23

**Authors:** Anke Schwarzer, Edwin Weber

**Affiliations:** a Institut für Organische Chemie, TU Bergakademie Freiberg, Leipziger Strasse 29, D-09596 Freiberg/Sachsen, Germany

## Abstract

In the title compound, C_23_H_8_F_10_O_2_, the three arene rings are twisted one with respect to the other: the two perfluorinated arene rings are tilted to each other by an angle of 60.39 (7)°. They are inclined to the non-fluorinated phenyl unit by 38.85 (7) and 78.74 (7)°. The olefinic double bond adopts an *E* configuration. The carbonyl groups are not in a coplanar alignment with reference to the neighbouring arene rings. The crystal packing features a number of weak C—H⋯F inter­actions, which leads to the formation of a three-dimensional network.

## Related literature

For a detailed discussion of fluorinated chalcones, see: Cesarin-Sobrinho *et al.* (2001 ▶); Cesarin-Sobrinho & Netto-Ferreira (2002 ▶).
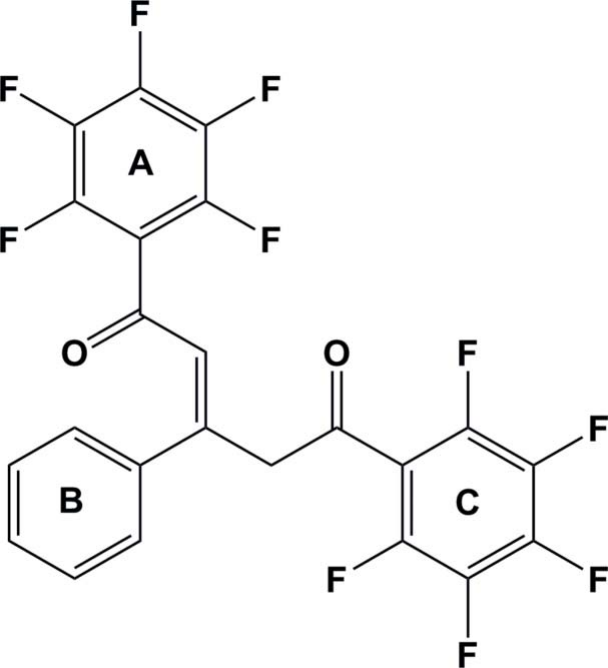


## Experimental

### 

#### Crystal data

C_23_H_8_F_10_O_2_
*M* *_r_* = 506.29
Monoclinic, 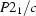

*a* = 10.9860 (3) Å
*b* = 15.8843 (4) Å
*c* = 11.4963 (3) Å
β = 101.528 (1)°
*V* = 1965.69 (9) Å^3^
*Z* = 4
Mo *K*α radiation
μ = 0.17 mm^−1^
*T* = 93 K
0.46 × 0.43 × 0.41 mm

#### Data collection

Bruker SMART CCD area-detector diffractometer
Absorption correction: none
18281 measured reflections
3657 independent reflections
3210 reflections with *I* > 2σ(*I*)
*R*_int_ = 0.019

#### Refinement

*R*[*F* ^2^ > 2σ(*F* ^2^)] = 0.029
*wR*(*F* ^2^) = 0.092
*S* = 1.03
3657 reflections
316 parameters
H-atom parameters constrained
Δρ_max_ = 0.28 e Å^−3^
Δρ_min_ = −0.20 e Å^−3^

### 

Data collection: *SMART* (Bruker, 2007 ▶); cell refinement: *SAINT* (Bruker, 2007 ▶); data reduction: *SAINT*; program(s) used to solve structure: *SHELXS97* (Sheldrick, 2008 ▶); program(s) used to refine structure: *SHELXL97* (Sheldrick, 2008 ▶); molecular graphics: *SHELXTL* (Sheldrick, 2008 ▶); software used to prepare material for publication: *SHELXTL*.

## Supplementary Material

Crystal structure: contains datablocks global, I. DOI: 10.1107/S1600536809041701/su2150sup1.cif

Structure factors: contains datablocks I. DOI: 10.1107/S1600536809041701/su2150Isup2.hkl

Additional supplementary materials:  crystallographic information; 3D view; checkCIF report

## Figures and Tables

**Table 1:** Hydrogen-bond geometry (Å, °)

*D*—H⋯*A*	*D*—H	H⋯*A*	*D*⋯*A*	*D*—H⋯*A*
C13—H13⋯F7^i^	0.95	2.54	3.4393 (17)	159
C16—H16*B*⋯F2^ii^	0.99	2.46	3.4004 (15)	159
C11—H11⋯F9^iii^	0.95	2.56	3.2326 (18)	128

Symmetry codes: (i) 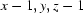; (ii) 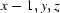; (iii) 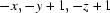.

**Fig. 1.**
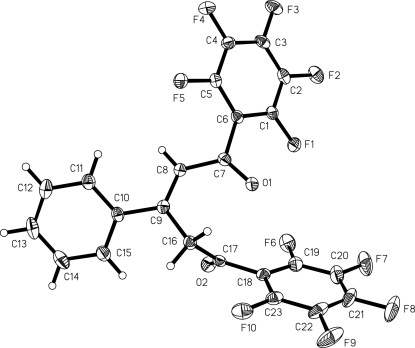
A view of the molecular structure of the title compound, showing 50% probability displacement ellipsoids.

**Fig. 2.**
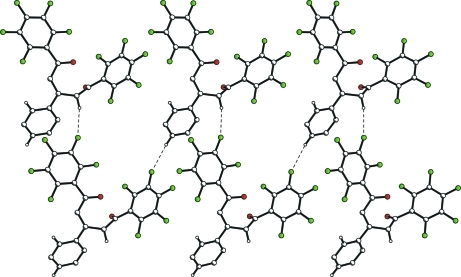
A partial view of the crystal packing, viewed along the b axis, of the title compound. C—H···F contacts as shown as broken lines - see Table 1 for details [Non-relevant H atoms have been omitted for clarity].

## supplementary crystallographic information

### Comment

The title compound (Fig. 1) exhibits a non-planar structure, which can be described by the dihedral and torsion angles of the arene rings and carbonyl groups with respect to one another. While the two perfluorinated arene rings A (C1-C6) and C (C18-C23) are tilted to each other at an angle of 60.39 (7) °, they are inclined to the non-fluorinated phenyl ring B (C10-C15) by 38.85 (7) and 78.74 (7)°, respectively. The carbonyl groups are tilted with reference to the adjacent perfluoro arene units, with torsion angles C1-C6-C7-O1 and O2-C17-C18-C19 being 37.76 (19)° and 43.27 (18) °, respectively. The olefinic double bond is fixed in the (*E*)-configuration.

In the crystal structure of the title compound adjacent molecules are linked by two C—H···F contacts (C13-H13···F7^i^ and C11-H11···F9^ii^), so forming a two dimensional network parallel to the ac plane [Table 1 and Fig. 2]. A third C-H···F interaction (C16-H16B···F2^iii^) links these planes to form a three dimensional network. Although two polar carbonyl groups are present in the molecule, their oxygen atoms are not involved in the formation of intermolecular contacts.

### Experimental

The title compound was obtained as a by-product during the synthesis of pentafluorochalcone from a 1:1 mixture of 2,3,4,5,6-pentafluoroacetophenone and benzaldehyde dissolved in sulfuric acid. After separation of the main product colourless single crystals were isolated from the filtrate on slow evaporation in air at rt.

### Refinement

The H-atoms were positioned geometrically and allowed to ride on their parent atoms: C—H = 0.95 - 0.99 Å with *U*_iso_ = 1.2*U*_eq_(parent C-atom).

### Crystal data

**Table:**

C_23_H_8_F_10_O_2_	*F*(000) = 1008
*M**_r_* = 506.29	*D*_x_ = 1.711 Mg m^−^^3^
Monoclinic, *P*2_1_/*c*	Mo *K*α radiation, λ = 0.71073 Å
Hall symbol: -P 2ybc	Cell parameters from 5070 reflections
*a* = 10.9860 (3) Å	θ = 3.1–36.1°
*b* = 15.8843 (4) Å	µ = 0.17 mm^−^^1^
*c* = 11.4963 (3) Å	*T* = 93 K
β = 101.528 (1)°	Plate, colourless
*V* = 1965.69 (9) Å^3^	0.46 × 0.43 × 0.41 mm
*Z* = 4	

### Data collection

**Table:**

Bruker SMART CCD area-detector diffractometer	3210 reflections with *I* > 2σ(*I*)
Radiation source: fine-focus sealed tube	*R*_int_ = 0.019
graphite	θ_max_ = 25.5°, θ_min_ = 1.9°
phi and ω scans	*h* = −13→13
18281 measured reflections	*k* = −19→17
3657 independent reflections	*l* = −13→13

### Refinement

**Table:**

Refinement on *F*^2^	Primary atom site location: structure-invariant direct methods
Least-squares matrix: full	Secondary atom site location: difference Fourier map
*R*[*F*^2^ > 2σ(*F*^2^)] = 0.029	Hydrogen site location: inferred from neighbouring sites
*wR*(*F*^2^) = 0.092	H-atom parameters constrained
*S* = 1.03	*w* = 1/[σ^2^(*F*_o_^2^) + (0.0587*P*)^2^ + 0.6558*P*] where *P* = (*F*_o_^2^ + 2*F*_c_^2^)/3
3657 reflections	(Δ/σ)_max_ < 0.001
316 parameters	Δρ_max_ = 0.28 e Å^−^^3^
0 restraints	Δρ_min_ = −0.20 e Å^−^^3^

### Special details

**Table:**

Geometry. All e.s.d.'s (except the e.s.d. in the dihedral angle between two l.s. planes) are estimated using the full covariance matrix. The cell e.s.d.'s are taken into account individually in the estimation of e.s.d.'s in distances, angles and torsion angles; correlations between e.s.d.'s in cell parameters are only used when they are defined by crystal symmetry. An approximate (isotropic) treatment of cell e.s.d.'s is used for estimating e.s.d.'s involving l.s. planes.
Refinement. Refinement of *F*^2^ against ALL reflections. The weighted *R*-factor *wR* and goodness of fit *S* are based on *F*^2^, conventional *R*-factors *R* are based on *F*, with *F* set to zero for negative *F*^2^. The threshold expression of *F*^2^ > σ(*F*^2^) is used only for calculating *R*-factors(gt) *etc*. and is not relevant to the choice of reflections for refinement. *R*-factors based on *F*^2^ are statistically about twice as large as those based on *F*, and *R*- factors based on ALL data will be even larger.

### Fractional atomic coordinates and isotropic or equivalent isotropic displacement parameters (Å^2^)

**Table:**

	*x*	*y*	*z*	*U*_iso_*/*U*_eq_	
O1	0.26093 (9)	0.41039 (7)	0.53299 (8)	0.0240 (2)	
O2	0.09160 (9)	0.23393 (6)	0.54477 (9)	0.0243 (2)	
F1	0.49682 (7)	0.36112 (5)	0.53619 (7)	0.0238 (2)	
F2	0.69999 (7)	0.39896 (6)	0.45459 (8)	0.0303 (2)	
F3	0.68309 (8)	0.48896 (6)	0.24995 (8)	0.0302 (2)	
F4	0.45871 (8)	0.54586 (6)	0.13327 (8)	0.0314 (2)	
F5	0.25233 (7)	0.50954 (6)	0.21465 (7)	0.0272 (2)	
F6	0.32482 (8)	0.25147 (6)	0.68712 (8)	0.0307 (2)	
F7	0.43936 (9)	0.29180 (7)	0.90694 (10)	0.0468 (3)	
F8	0.32401 (12)	0.38979 (7)	1.04497 (8)	0.0531 (3)	
F9	0.08804 (12)	0.44422 (6)	0.96211 (8)	0.0453 (3)	
F10	−0.02794 (8)	0.40693 (6)	0.73839 (8)	0.0308 (2)	
C1	0.48305 (12)	0.40581 (8)	0.43569 (12)	0.0184 (3)	
C2	0.58917 (12)	0.42447 (9)	0.39365 (13)	0.0211 (3)	
C3	0.58098 (12)	0.47069 (9)	0.29088 (13)	0.0220 (3)	
C4	0.46655 (13)	0.49898 (9)	0.23136 (12)	0.0218 (3)	
C5	0.36102 (12)	0.47898 (9)	0.27382 (12)	0.0196 (3)	
C6	0.36584 (12)	0.43131 (8)	0.37599 (12)	0.0178 (3)	
C7	0.25245 (12)	0.40885 (8)	0.42556 (12)	0.0187 (3)	
C8	0.14008 (12)	0.38285 (8)	0.34137 (12)	0.0184 (3)	
H8	0.1444	0.3779	0.2599	0.022*	
C9	0.03089 (12)	0.36557 (8)	0.37313 (12)	0.0173 (3)	
C10	−0.07770 (12)	0.34003 (9)	0.28121 (12)	0.0186 (3)	
C11	−0.09786 (12)	0.37620 (9)	0.16798 (12)	0.0216 (3)	
H11	−0.0407	0.4165	0.1495	0.026*	
C12	−0.20054 (14)	0.35365 (10)	0.08255 (13)	0.0277 (3)	
H12	−0.2144	0.3793	0.0064	0.033*	
C13	−0.28304 (14)	0.29369 (11)	0.10805 (15)	0.0340 (4)	
H13	−0.3531	0.2780	0.0493	0.041*	
C14	−0.26309 (14)	0.25668 (11)	0.21924 (16)	0.0346 (4)	
H14	−0.3192	0.2151	0.2362	0.042*	
C15	−0.16197 (13)	0.27982 (10)	0.30601 (14)	0.0256 (3)	
H15	−0.1499	0.2548	0.3825	0.031*	
C16	0.01318 (12)	0.37282 (9)	0.50062 (12)	0.0187 (3)	
H16A	0.0422	0.4289	0.5322	0.022*	
H16B	−0.0764	0.3683	0.5019	0.022*	
C17	0.08369 (11)	0.30502 (8)	0.58018 (12)	0.0176 (3)	
C18	0.14383 (12)	0.32769 (8)	0.70533 (12)	0.0191 (3)	
C19	0.26301 (13)	0.29878 (9)	0.75257 (13)	0.0234 (3)	
C20	0.32346 (15)	0.31974 (10)	0.86547 (14)	0.0310 (4)	
C23	0.08752 (13)	0.37790 (9)	0.77799 (13)	0.0234 (3)	
C22	0.14702 (17)	0.39821 (9)	0.89257 (13)	0.0312 (4)	
C21	0.26522 (17)	0.36956 (10)	0.93524 (13)	0.0345 (4)	

### Atomic displacement parameters (Å^2^)

**Table:**

	*U*^11^	*U*^22^	*U*^33^	*U*^12^	*U*^13^	*U*^23^
O1	0.0200 (5)	0.0339 (6)	0.0175 (5)	−0.0065 (4)	0.0026 (4)	−0.0014 (4)
O2	0.0289 (5)	0.0193 (5)	0.0244 (5)	0.0001 (4)	0.0045 (4)	−0.0027 (4)
F1	0.0226 (4)	0.0249 (4)	0.0223 (4)	0.0012 (3)	0.0007 (3)	0.0039 (3)
F2	0.0149 (4)	0.0330 (5)	0.0416 (5)	0.0055 (3)	0.0023 (3)	0.0001 (4)
F3	0.0225 (4)	0.0350 (5)	0.0374 (5)	−0.0061 (4)	0.0164 (4)	−0.0056 (4)
F4	0.0339 (5)	0.0360 (5)	0.0254 (5)	−0.0083 (4)	0.0086 (4)	0.0075 (4)
F5	0.0192 (4)	0.0343 (5)	0.0262 (4)	0.0008 (3)	0.0003 (3)	0.0086 (4)
F6	0.0234 (4)	0.0285 (5)	0.0384 (5)	0.0074 (3)	0.0019 (4)	0.0002 (4)
F7	0.0413 (6)	0.0345 (6)	0.0506 (6)	−0.0048 (4)	−0.0248 (5)	0.0138 (5)
F8	0.0928 (9)	0.0368 (6)	0.0194 (5)	−0.0223 (6)	−0.0137 (5)	0.0026 (4)
F9	0.0897 (8)	0.0268 (5)	0.0263 (5)	−0.0026 (5)	0.0279 (5)	−0.0046 (4)
F10	0.0317 (5)	0.0309 (5)	0.0343 (5)	0.0057 (4)	0.0172 (4)	−0.0005 (4)
C1	0.0190 (7)	0.0165 (7)	0.0191 (7)	−0.0013 (5)	0.0024 (5)	−0.0026 (5)
C2	0.0155 (6)	0.0198 (7)	0.0271 (7)	0.0012 (5)	0.0023 (5)	−0.0067 (6)
C3	0.0192 (7)	0.0221 (7)	0.0276 (8)	−0.0051 (5)	0.0116 (6)	−0.0080 (6)
C4	0.0265 (7)	0.0213 (7)	0.0182 (7)	−0.0052 (5)	0.0060 (6)	−0.0010 (5)
C5	0.0169 (6)	0.0214 (7)	0.0194 (7)	−0.0009 (5)	0.0005 (5)	−0.0021 (5)
C6	0.0170 (6)	0.0180 (7)	0.0184 (7)	−0.0021 (5)	0.0036 (5)	−0.0038 (5)
C7	0.0177 (6)	0.0177 (7)	0.0206 (7)	−0.0008 (5)	0.0037 (5)	0.0003 (5)
C8	0.0183 (6)	0.0196 (7)	0.0167 (7)	−0.0009 (5)	0.0024 (5)	0.0001 (5)
C9	0.0183 (6)	0.0145 (6)	0.0183 (7)	0.0023 (5)	0.0016 (5)	0.0009 (5)
C10	0.0149 (6)	0.0197 (7)	0.0206 (7)	0.0024 (5)	0.0019 (5)	−0.0030 (5)
C11	0.0190 (6)	0.0232 (7)	0.0219 (7)	0.0023 (5)	0.0024 (5)	−0.0022 (5)
C12	0.0268 (8)	0.0315 (8)	0.0217 (7)	0.0067 (6)	−0.0030 (6)	−0.0048 (6)
C13	0.0223 (7)	0.0379 (9)	0.0358 (9)	0.0000 (6)	−0.0083 (6)	−0.0116 (7)
C14	0.0231 (8)	0.0336 (9)	0.0449 (10)	−0.0102 (6)	0.0014 (7)	−0.0044 (7)
C15	0.0216 (7)	0.0266 (8)	0.0275 (8)	−0.0029 (6)	0.0025 (6)	0.0010 (6)
C16	0.0155 (6)	0.0212 (7)	0.0192 (7)	0.0008 (5)	0.0032 (5)	−0.0014 (5)
C17	0.0141 (6)	0.0201 (7)	0.0196 (7)	−0.0014 (5)	0.0062 (5)	−0.0006 (5)
C18	0.0232 (7)	0.0165 (7)	0.0179 (7)	−0.0032 (5)	0.0049 (5)	0.0021 (5)
C19	0.0258 (7)	0.0181 (7)	0.0250 (8)	−0.0019 (5)	0.0016 (6)	0.0038 (6)
C20	0.0342 (8)	0.0221 (8)	0.0301 (8)	−0.0071 (6)	−0.0094 (6)	0.0096 (6)
C23	0.0301 (7)	0.0187 (7)	0.0232 (7)	−0.0025 (6)	0.0096 (6)	0.0032 (6)
C22	0.0599 (11)	0.0179 (7)	0.0189 (7)	−0.0071 (7)	0.0154 (7)	−0.0008 (6)
C21	0.0597 (11)	0.0236 (8)	0.0150 (7)	−0.0159 (7)	−0.0044 (7)	0.0044 (6)

### Geometric parameters (Å, °)

**Table:**

O1—C7	1.2202 (17)	C9—C16	1.5210 (18)
O2—C17	1.2095 (17)	C10—C11	1.399 (2)
F1—C1	1.3386 (16)	C10—C15	1.399 (2)
F2—C2	1.3403 (16)	C11—C12	1.386 (2)
F3—C3	1.3322 (15)	C11—H11	0.9500
F4—C4	1.3397 (16)	C12—C13	1.386 (2)
F5—C5	1.3415 (16)	C12—H12	0.9500
F6—C19	1.3403 (17)	C13—C14	1.384 (2)
F7—C20	1.3427 (19)	C13—H13	0.9500
F8—C21	1.3365 (18)	C14—C15	1.386 (2)
F9—C22	1.3416 (18)	C14—H14	0.9500
F10—C23	1.3409 (17)	C15—H15	0.9500
C1—C2	1.3805 (19)	C16—C17	1.5208 (18)
C1—C6	1.3928 (19)	C16—H16A	0.9900
C2—C3	1.379 (2)	C16—H16B	0.9900
C3—C4	1.381 (2)	C17—C18	1.5021 (18)
C4—C5	1.3818 (19)	C18—C23	1.387 (2)
C5—C6	1.3896 (19)	C18—C19	1.391 (2)
C6—C7	1.5125 (18)	C19—C20	1.375 (2)
C7—C8	1.4676 (18)	C20—C21	1.373 (3)
C8—C9	1.3500 (19)	C23—C22	1.386 (2)
C8—H8	0.9500	C22—C21	1.371 (3)
C9—C10	1.4834 (18)		
			
F1—C1—C2	117.28 (12)	C14—C13—C12	119.85 (14)
F1—C1—C6	120.89 (12)	C14—C13—H13	120.1
C2—C1—C6	121.83 (13)	C12—C13—H13	120.1
F2—C2—C3	120.39 (12)	C13—C14—C15	120.54 (15)
F2—C2—C1	119.64 (13)	C13—C14—H14	119.7
C3—C2—C1	119.96 (13)	C15—C14—H14	119.7
F3—C3—C2	120.22 (13)	C14—C15—C10	120.15 (14)
F3—C3—C4	120.10 (13)	C14—C15—H15	119.9
C2—C3—C4	119.67 (12)	C10—C15—H15	119.9
F4—C4—C3	119.80 (12)	C17—C16—C9	112.21 (11)
F4—C4—C5	120.52 (13)	C17—C16—H16A	109.2
C3—C4—C5	119.68 (13)	C9—C16—H16A	109.2
F5—C5—C4	117.54 (12)	C17—C16—H16B	109.2
F5—C5—C6	120.36 (12)	C9—C16—H16B	109.2
C4—C5—C6	122.07 (13)	H16A—C16—H16B	107.9
C5—C6—C1	116.76 (12)	O2—C17—C18	119.92 (12)
C5—C6—C7	123.49 (12)	O2—C17—C16	121.40 (12)
C1—C6—C7	119.72 (12)	C18—C17—C16	118.68 (11)
O1—C7—C8	123.86 (12)	C23—C18—C19	116.93 (13)
O1—C7—C6	118.34 (12)	C23—C18—C17	123.44 (12)
C8—C7—C6	117.74 (11)	C19—C18—C17	119.62 (12)
C9—C8—C7	123.69 (12)	F6—C19—C20	117.53 (13)
C9—C8—H8	118.2	F6—C19—C18	120.58 (12)
C7—C8—H8	118.2	C20—C19—C18	121.85 (14)
C8—C9—C10	119.63 (12)	F7—C20—C21	120.18 (14)
C8—C9—C16	122.47 (12)	F7—C20—C19	119.99 (16)
C10—C9—C16	117.88 (11)	C21—C20—C19	119.83 (15)
C11—C10—C15	118.81 (12)	F10—C23—C22	118.49 (13)
C11—C10—C9	120.37 (12)	F10—C23—C18	119.92 (13)
C15—C10—C9	120.82 (12)	C22—C23—C18	121.58 (14)
C12—C11—C10	120.53 (14)	F9—C22—C21	120.22 (15)
C12—C11—H11	119.7	F9—C22—C23	120.05 (16)
C10—C11—H11	119.7	C21—C22—C23	119.72 (15)
C13—C12—C11	120.11 (14)	F8—C21—C22	120.16 (16)
C13—C12—H12	119.9	F8—C21—C20	119.76 (16)
C11—C12—H12	119.9	C22—C21—C20	120.07 (14)
			
F1—C1—C2—F2	−1.07 (19)	C10—C11—C12—C13	−1.2 (2)
C6—C1—C2—F2	179.64 (12)	C11—C12—C13—C14	0.4 (2)
F1—C1—C2—C3	−179.66 (12)	C12—C13—C14—C15	0.8 (3)
C6—C1—C2—C3	1.0 (2)	C13—C14—C15—C10	−1.1 (2)
F2—C2—C3—F3	1.4 (2)	C11—C10—C15—C14	0.2 (2)
C1—C2—C3—F3	179.93 (12)	C9—C10—C15—C14	179.92 (14)
F2—C2—C3—C4	−177.77 (12)	C8—C9—C16—C17	−69.80 (16)
C1—C2—C3—C4	0.8 (2)	C10—C9—C16—C17	111.38 (13)
F3—C3—C4—F4	−1.0 (2)	C9—C16—C17—O2	−37.89 (17)
C2—C3—C4—F4	178.17 (12)	C9—C16—C17—C18	142.37 (12)
F3—C3—C4—C5	179.33 (12)	O2—C17—C18—C23	−137.78 (14)
C2—C3—C4—C5	−1.5 (2)	C16—C17—C18—C23	41.95 (18)
F4—C4—C5—F5	−1.3 (2)	O2—C17—C18—C19	43.27 (18)
C3—C4—C5—F5	178.44 (12)	C16—C17—C18—C19	−136.99 (13)
F4—C4—C5—C6	−179.26 (12)	C23—C18—C19—F6	−178.52 (12)
C3—C4—C5—C6	0.5 (2)	C17—C18—C19—F6	0.49 (19)
F5—C5—C6—C1	−176.62 (12)	C23—C18—C19—C20	−0.9 (2)
C4—C5—C6—C1	1.3 (2)	C17—C18—C19—C20	178.13 (13)
F5—C5—C6—C7	1.5 (2)	F6—C19—C20—F7	−1.1 (2)
C4—C5—C6—C7	179.47 (13)	C18—C19—C20—F7	−178.76 (13)
F1—C1—C6—C5	178.66 (12)	F6—C19—C20—C21	178.63 (13)
C2—C1—C6—C5	−2.1 (2)	C18—C19—C20—C21	0.9 (2)
F1—C1—C6—C7	0.43 (19)	C19—C18—C23—F10	−179.14 (12)
C2—C1—C6—C7	179.71 (12)	C17—C18—C23—F10	1.9 (2)
C5—C6—C7—O1	−140.34 (14)	C19—C18—C23—C22	−0.2 (2)
C1—C6—C7—O1	37.76 (19)	C17—C18—C23—C22	−179.15 (13)
C5—C6—C7—C8	42.30 (19)	F10—C23—C22—F9	1.6 (2)
C1—C6—C7—C8	−139.60 (13)	C18—C23—C22—F9	−177.41 (13)
O1—C7—C8—C9	6.5 (2)	F10—C23—C22—C21	−179.82 (13)
C6—C7—C8—C9	−176.30 (13)	C18—C23—C22—C21	1.2 (2)
C7—C8—C9—C10	179.78 (12)	F9—C22—C21—F8	−2.3 (2)
C7—C8—C9—C16	1.0 (2)	C23—C22—C21—F8	179.05 (13)
C8—C9—C10—C11	−36.74 (19)	F9—C22—C21—C20	177.44 (14)
C16—C9—C10—C11	142.12 (13)	C23—C22—C21—C20	−1.2 (2)
C8—C9—C10—C15	143.55 (14)	F7—C20—C21—F8	−0.4 (2)
C16—C9—C10—C15	−37.59 (18)	C19—C20—C21—F8	179.91 (13)
C15—C10—C11—C12	0.9 (2)	F7—C20—C21—C22	179.82 (13)
C9—C10—C11—C12	−178.77 (13)	C19—C20—C21—C22	0.1 (2)

### Hydrogen-bond geometry (Å, °)

**Table:**

*D*—H···*A*	*D*—H	H···*A*	*D*···*A*	*D*—H···*A*
C13—H13···F7^i^	0.95	2.54	3.4393 (17)	159
C16—H16B···F2^ii^	0.99	2.46	3.4004 (15)	159
C11—H11···F9^iii^	0.95	2.56	3.2326 (18)	128

Symmetry codes: (i) *x*−1, *y*, *z*−1; (ii) *x*−1, *y*, *z*; (iii) −*x*, −*y*+1, −*z*+1.
